# Reporting changes in right ventricular systolic pressure: insights from Classification and Regression Tree (CART) analysis

**DOI:** 10.1186/s44156-026-00120-8

**Published:** 2026-06-08

**Authors:** Shadi P. Bagherzadeh, Kartik S. Malunjkar, Neha M. Mantri, Bettia E. Celestin, Roham T. Zamanian, Paul Heidenreich, Ingela Schnittger, Michael Salerno, David Oxborough, Daniel Augustine, Raymond Benza, Everton J. Santana, Francois Haddad

**Affiliations:** 1 Division of Cardiovascular Medicine, Department of Medicine, Stanford University, Palo Alto, CA USA; 2 Stanford Cardiovascular Institute, Palo Alto, CA USA; 3 Veteran Affair Healthcare Systems, Palo Alto, CA USA; 4 Stanford Division of Pulmonary, Allergy and Critical Care Medicine, Department of Medicine, Stanford University, Palo Alto, CA USA; 5 Liverpool John Moores University, Liverpool, UK; 6 Royal United Hospital, Bath, UK; 7 Mount Sinai Health Systems, New York, NY USA; 8 Research Unit Hypertension and Cardiovascular Epidemiology, Leuven Department of Cardiovascular Sciences, University of Leuven, Leuven, KU Belgium

**Keywords:** Pulmonary hypertension, Right ventricular systolic pressure, CART analysis, Reference change, Analytical variability

## Abstract

**Background:**

Right ventricular systolic pressure (RVSP) is an echocardiographic metric to monitor pulmonary hypertension (PH). However, there is no recommendation on what constitutes a meaningful change. In this study, we aimed to gain insight into how physicians at our institution report significant changes in RVSP. We then aimed to quantify the analytic variability of reported RVSP using duplicate analysis.

**Methods:**

We utilized the Stanford CardioShare Registry to identify 5,934 patients with 32,656 echocardiogram pairs with reported RVSP. Natural Language Processing was employed to categorize pairs into decrease, increase, no change, and no direct mention. Classification and Regression Tree (CART) analysis was applied to these groups to identify reporting thresholds among physicians. To assess the performance of the CART model, accuracy, precision, recall, and F1-score were reported using a stratified cross-validation method. In a separate cohort comprising 210 healthy volunteers and 208 patients with PH, two blinded core laboratory cardiologists measured the peak tricuspid regurgitation velocity. We employed a duplicate analysis method to model bias and a robust precision method for reporting RVSP and assessing analytical variability.

**Results:**

Of the total pairs of echocardiographic studies, RVSP was reported as stable in 48.9%, increased in 12.5%, decreased in 9.9% while 28.7% did not have a direct reference to RVSP change. CART analysis revealed that physicians most commonly determine change based on an absolute threshold of 8 mmHg and whether the change occurred within or outside the reference range. On cross-validation, the accuracy and F1-score were 83% and 79% for the increase and 81% and 74% for the decrease algorithms. In the duplicate analysis cohort, the analytic precision was 8–10% with worst relative precision at lower values of RVSP. This translates into a 15% reference change value, assuming a 4–5% biological variation.

**Conclusion:**

The study provides insights on real world practice of physician reporting in RVSP and provides directions for future recommendations regarding report changes.

**Supplementary Information:**

The online version contains supplementary material available at 10.1186/s44156-026-00120-8.

## Background

Pulmonary hypertension (PH) is recognized as a significant predictor of outcomes in cardiovascular and pulmonary diseases [1]. According to the European Society of Cardiology and the European Respiratory Society (ESC/ERS), PH is defined with a right heart catheterization by a mean pulmonary arterial pressure (MPAP) exceeding 20 mmHg [1], which generally corresponds to a pulmonary arterial systolic pressure (PASP) ranging from 30 to 35 mmHg [2]. Transthoracic echocardiography (TTE) plays a central role in assessing and monitoring PH [1, 2]. PASP is commonly estimated using right ventricular systolic pressure (RVSP) in the absence of right ventricular outflow tract obstruction. RVSP is an essential echocardiographic parameter in monitoring patients with heart failure and assessing response to therapy in patients with PH [3–6].

Although RVSP is an essential parameter in clinical practice [7], there are currently no guidelines on how to report changes in RVSP. In laboratory medicine, the reference change value (RCV) is used to establish absolute or relative thresholds for reporting changes in measured values, and it plays a crucial role in subsequent patient management [8–11]. These thresholds account for technical (analytic) as well as biological variability. Analytic variability refers to differences in measurements between readers or instruments; for RVSP these can be secondary to differences in Doppler insonation angle, the choice of interface for peak velocity estimates (modal or non-modal), or short-term variability related to respiratory-phasic changes. In addition, technical variability associated with the assumptions of the Bernoulli equation could also contribute to variability [6]. In contrast, biological variability is observed over longer periods of time and reflects changes associated with hemodynamics, physiological states, or circadian rhythms.

Clinicians often rely on their experience to interpret and determine what represents meaningful change. Statistical methods have recently been developed to identify thresholds more influential for classification or regression tasks. One such method is the Classification and Regression Tree (CART) analysis, which identifies thresholds based on the Gini index, a measure of data impurity or variability [12, 13]. CART analysis has been used in imaging studies, for example, to determine how imaging results can complement clinical risk scores in heart failure [12]. Additionally, CART can help uncover thresholds that influence decision-making. [12, 13].

In our study, we aimed to investigate how physicians report changes in RVSP using CART analysis. We hypothesized that their decisions are influenced by both absolute change values and whether these changes fall within reference limits. Additionally, we evaluated whether these reporting thresholds align with the analytic variability of RVSP measurements. Based on our findings, we propose a practical approach for reporting changes in RVSP, which can later be tested in the context of clinically meaningful decision-making. The study is intended to provide insights on decision making and to invite future studies that will guide meaningful change in practice based on technical, biological, and outcome-driven thresholds.

## Method and study design

The study includes two parts: in the first part, we use CART-based analysis on paired echocardiographic studies of an echocardiographic registry, and the second part quantifies technical variability on duplicate analysis of echocardiographic studies by two blinded readers.

## Patient population selection for the rcv reporting analysis

### Part 1. Stanford cardioshare registry

The study was approved by the Stanford University Institutional Review Board (IRB#25673) as part of the CardioShare registry protocol, which includes right heart catheterization (RHC), echocardiography, and clinical data acquisition. To enrich the study of patients with advanced heart and lung disease, we focused on patients who underwent RHC with an indication at Stanford Healthcare Cath Lab. Retrospective text reports from digitized data of patients who underwent echocardiography and RHC conducted at Stanford Healthcare Center were considered.

#### RHC and echocardiography cohort selection

**Fig. 1:**
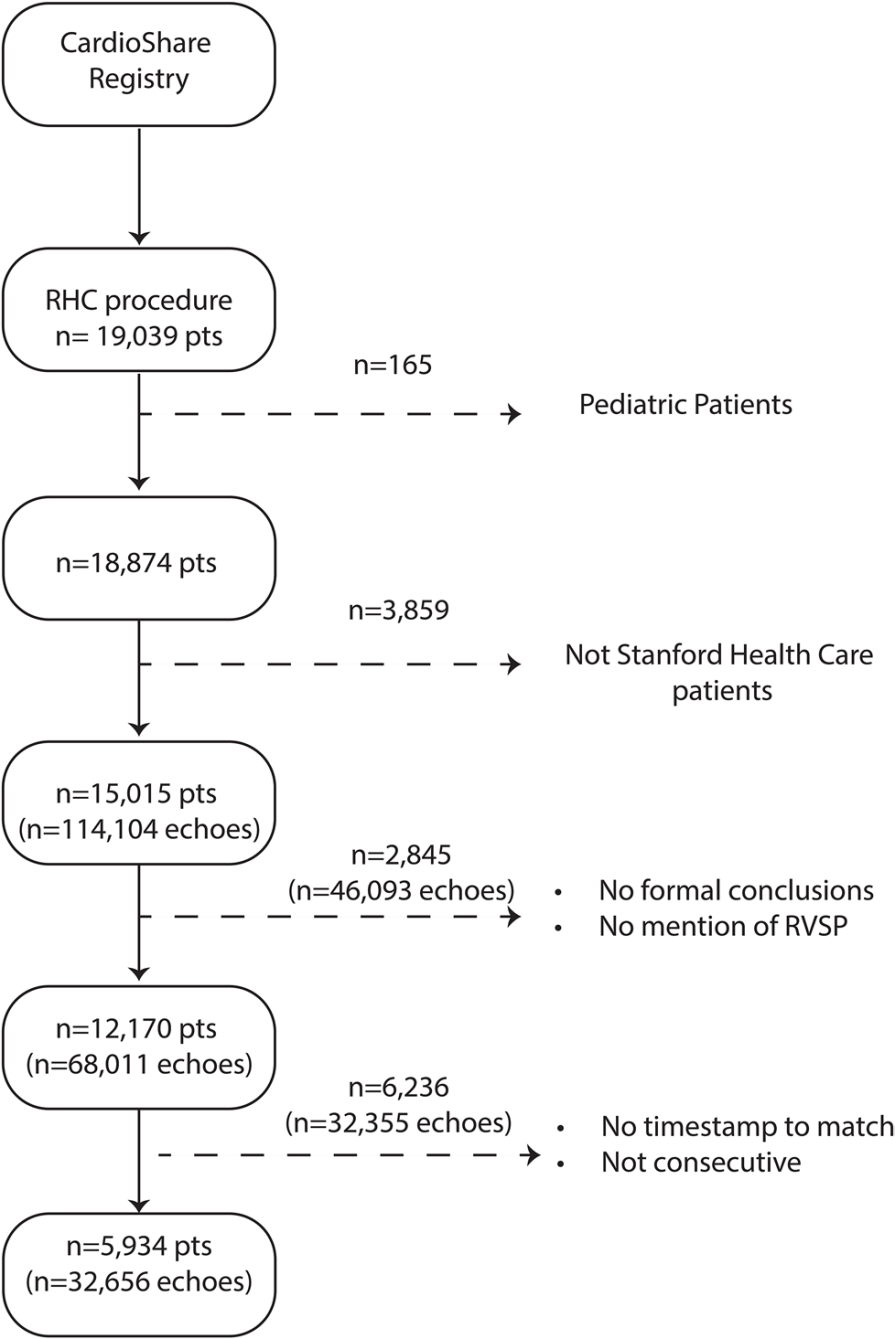
Flow chart demonstrating the cohort selection and study protocol from the CardioShare registry. n: numbers; pts: patients; RHC: right heart catheterization; RVSP: right ventricular systolic pressure

Between January 2005 and June 2023, electronic documents of 19,039 patients who were referred for RHC have been identified. RHC attributed to pediatric patients has been excluded. The echocardiography linkage database was created using a three-step process. We used Structured Query Language (SQL) language to extract the needed data by writing a query from Echo/Xcelera (version R3.1, Philips Healthcare, Amsterdam, The Netherlands) source (extract). We completed data standardization and quality control by removing duplicates and handling nulls (transform). Finally, we consolidated the data for analysis by integrating clinical and echocardiographic data. The target database was mapped to discrete quantitative variables, as well as interpretation and conclusion report fields. After linking RHC to the echocardiography database, 15,015 patients remained in the cohort with corresponding 114,104 transthoracic echocardiogram (TTE) reports. We did not include reports attributed to transesophageal echoes, vascular or abdominal duplex ultrasounds, or stress echo reports. After excluding patients without a formal TTE conclusion or no mention of RVSP in the report, 12,170 patients remained in the cohort, corresponding to 68,011 reports. Among them, TTE reports without a timestamp linking two studies were excluded. The final cohort consisted of 5,934 patients, corresponding to 32,656 echocardiograms with consecutive RVSP reports. Figure 1 summarizes the study cohort selection. For this study, we utilized electronic records from Cath Lab documentation, including measurements of mean pulmonary arterial pressure (MPAP), right atrial pressure (RAP), and cardiac output (CO) obtained using the assumed Fick method. Pulmonary vascular resistance (PVR) was calculated as (MPAP-RAP)/CO. The cardiac index (CI) was calculated as CO/body surface area. The extracted TTE metrics included RVSP, peak tricuspid regurgitation velocity (TRV_max_), and estimated right atrial pressure (eRAP) fields. All the above metrics had been measured according to the latest American Society of Echocardiography (ASE) guidelines [14, 15]. The most updated ASE and ESC/ERS guidelines definitions were used to re-estimate RAP and re-grade TR severity [1, 14].

### Part 2. Cohort for the analytic variability

For the duplicate analysis, we considered a separate cohort of healthy controls and patients who had a confirmed diagnosis of pulmonary arterial hypertension (PAH). The healthy cohort was prospectively recruited as part of the Stanford Aging Study, which included 210 participants with baseline TTEs. We retrospectively selected 208 patients having MPAP > 20 mmHg and pulmonary arterial wedge pressure (PAWP) < 15 mmHg. The echocardiograms were acquired using a Philips iE33 and EPIQ 7 model (Andover, MA, USA). The images were read blindly by two level 3 core laboratory (CL) cardiologists (FH, BC). Before analysis, the readers underwent a training phase, in which the reading methodology was standardized, including quality assessment and modal frequency assessment. Both CL readers used a Likert scale to quantify the quality of the Doppler signal with the following interpretation: grade 5: excellent; grade 4: good diagnostic quality; grade 3: sub-optimal but still interpretable; grade 2: interpretable with caution, and grade 1: non-interpretable. Gradation was also simplified to 3 groups for analysis after merging groups 4 and 5 (good), 3 (moderate), and 1 and 2 (low) (Additional File 1).

## Statistical analysis

### Part 1. RVSP reporting and CART analysis

#### Data extraction and classification of the reports

Report conclusions were analyzed using the Python Regular Expression (regex) module. The conclusion statement was manually curated in 250 cases, confirming the correct classification. After identifying the comparative statement, we classified the conclusions based on the comparative statement (last sentence) that contains keywords such as “compared to,” “in comparison,” and a specific timestamp (date). To classify each patient into specific categories, such as “no change,” “change increase,” “change decrease,” and “indeterminate,” a word bank of descriptor words commonly used by clinicians was created. If no significant changes were reported for all TTE metrics, the conclusion was classified as “no change.” If any metric other than RVSP was stated to change but RVSP was not mentioned, or the echocardiography was done for another reason, the conclusion was classified as “no clear mention” or “indeterminate.” If RVSP was stated to increase or decrease, they were classified as “increase” or “decrease,” respectively. If a status change was stated only corresponding to post-transplant or post-surgery, the pairs of TTEs were excluded. To ensure quality control, the qualitative statements “increase” and “decrease” were confirmed against the numerical increase or decrease.

#### Quantitative changes

For each pair of longitudinal RVSP measurements by echocardiography, we first calculated their absolute and relative changes. The absolute change was computed as the difference between follow-up and baseline RVSP, and the relative change was calculated as the ratio between the absolute change and the baseline RVSP expressed as a percentage.

#### CART analysis and gini index

CART algorithms were explored to identify the rules for the classifications. These classifiers were implemented using the scikit-learn (1.3.0) default configuration except for the maximum depth of the decision tree, which was set to 3. The depth of the decision tree refers to the number of splits from the root node (first decision) to the deepest leaf (final decision). For classification tasks, CART uses the Gini Index (Gini Impurity or Gini coefficient) to measure the degree of purity or impurity of a data set at each node. The Gini Index is calculated as Gini (*p*_*i*_) = 1- ∑ p_i_^2^, where *p*_*i*_ is the probability of an item being classified into a particular class. During the construction of the decision tree, the split that results in the smallest Gini Index is selected. The closer the Gini Index to 0 indicates less impurity, whereas the Gini Index of 0.5 indicates a maximum impurity (50% of the data belong to the two decision classes).

For change classifications, clinicians can consider absolute or relative change and be influenced by the baseline or the current RVSP value. Because of associations between change measures and values, we selected three values for splitting branches (absolute, relative change, and either baseline or follow-up value). The CART analysis built two distinct binary trees, as putting all into one would lose insight into reference change. One compares “increase” with “no change” while the other compares “decrease” and “no change” categories.

#### Decision tree performance evaluation

We applied the stratified cross-validation method to validate the model’s prediction and performance against the actual outcome. After splitting data into five folds, we trained the model on the four derivation sets and validated the performance on the testing set. Model performance metrics, including accuracy, precision, recall, and the F1-score, were computed on the testing set. Accuracy is calculated as TP + TN/ (n), where TP is the true positive, TN is the true negative, and n is the total number. Precision is calculated as TP/(TP + FP) and recalled as TP /(TP + FN), where FP is false positive, and FN is false negative. Finally, the F1-score, as a balanced metric between precision and recall, was calculated as 2*(Precision * Recall)/ (Precision + Recall).

#### Descriptive statistics

Quantitative values are presented using mean and standard deviation (SD) if normal distribution or median and percentile otherwise. Counts are presented as numbers (n/N) and percentages (%). Comparisons between groups were conducted through the Mann-Whitney U test. A P-value(P) less than 0.01 was employed as significance. For all the analysis, Python 3.11.6 libraries and modules were employed.

### Part 2. Analytical variability and modeling of RCV

RVSP was calculated according to the Bernoulli equation using 4* peak tricuspid regurgitation velocity (TRV)^2^ + estimated right atrial pressure (RAP). Several measures of differences are reported. First, the absolute difference between the two readers is reported as reader 1 – reader 2. Differences were then scaled to the square root of 2 (√2) to account for their arising from the imprecision of two measures. Percentile precision was then calculated using a robust percentile method as half of the 84th and 16th difference (CVa) [20]. The reference change incorporates both the analytic and the within-subject variation as RCV = Z * √2 * √ (Cva^2^ + Cvi^2^ ). For the biological variability, we assume a very conservative 4% biological variability based on longitudinal studies using CardioMEMS in patients with heart failure [16].

## Results

### Part 1. Result of cardio-share cath lab registry

#### Descriptive analysis

The characteristics of the cohort selected from the CardioShare registry, including the demographics, baseline echocardiographic measurements, and invasively measured RHC pressures, are shown in Table 1. The mean time difference between RHC and echocardiography was 4.13 years (±2.8). TTE and RHC studies more than 7.8 years apart have not been included. The analysis focused on paired echocardiographic studies. Most had preserved left ventricular ejection fraction (LVEF), with the majority meeting the hemodynamic criteria for pulmonary hypertension (PH), according to the most recent ESC/ERS guidelines for PH [1]. The median RVSP was 41.07 [22.60–100.30] mmHg in patients having LVEF > 52%, 38.85 [19.66–88.50] mmHg in the 41%< LVEF < 51% category, 40.46 [20.55–85.20]mmHg in the 30%< LVEF < 40% group, and 44.08 [15.22–104.25] mmHg in the LVEF < 30% category [14].

**Table 1:** Demographics, hemodynamics, and basic echocardiography characteristics (when present) of patient from CardioShare registry

	Value (numerical: median [IQR], categorical: *n*, %)
**Patients Demographics**	
age (years)	62.0 [48.0–75.0]
sex (male)	3,145 (53%)
height (cm)	167.6 [160.0–175.3]
weight (kg)	74.8 [63.5–88.0]
**Echocardiographic metrics (n= data present)**	
LVID (cm) (*n* = 32,086)	4.6 [4.1–5.1]
LVEF (%) (*n* = 24,292)	58.4 [47.0–64.0]
RVIDd (cm) (*n* = 2,372)	3.3 [2.8–3.8]
TAPSE (cm) (*n* = 5,015)	1.8 [1.5–2.1]
Septal e’ (cm/s) (*n* = 11,766)	6.1 [4.9–7.6]
Lateral e’ (cm/s) (*n* = 12,055)	9.1 [7.1–11.5]
Average E/e’ (*n* = 11,652)	10.7 [8.2–14.4]
LAVI (mL/m^2^) (*n* = 14,393)	36.1 [26.6–49.7]
TR severity (TR) (cm/s) (*n* = 32,656)	Mild: 57.3% Mild-Moderate: 18.6% Moderate: 15.8% Moderately Severe: 4.1% Severe (3.1%)
RVSP (mmHg) (*n* = 32,656)	37.8 [29.3–52.0]
RAP (mmHg) (*n* = 32,656)	5.0 [5.0–10.0]
**Right heart catheterization measurements (patients)**	
SBP (mmHg)	133.0 [116.0–149.0]
DBP (mmHg)	73.0 [64.0–83.0]
HR (bpm)	75.0 [65.0–86.0]
Cardiac output (L/min)	4.7 [3.8–6.0]
Cardiac index (L/min/m^2^)	2.5 [2.1–3.1]
MPAP (mmHg)	25.0 [17.5–36.0]
PCWP (mmHg)	12.0 [8.0–17.0]
PVR (Woods Units)	2.2 [1.3–4.1]
MPAP > 20 mmHg	(62.9%)
PCWP > 15 mmHg	(30.3%)

*N = 32,656 samples corresponding to 5,934 patients

DBP: diastolic blood pressure; HR: heart rate; LAVI: left atrial volume index; LVEF: left ventricular ejection fraction; LVID: left ventricular internal diameter; MPAP: mean pulmonary arterial pressure; PCWP: pulmonary capillary wedge pressure; PVR: pulmonary vascular resistance; SBP: systolic blood pressure; RA: right atrium; RAP: right atrial pressure; RVDd: right ventricular diameter in diastole; TAPSE: tricuspid annular plane systolic excursion; TRV: tricuspid regurgitation

#### Reporting RVSP change

**Fig. 2:**
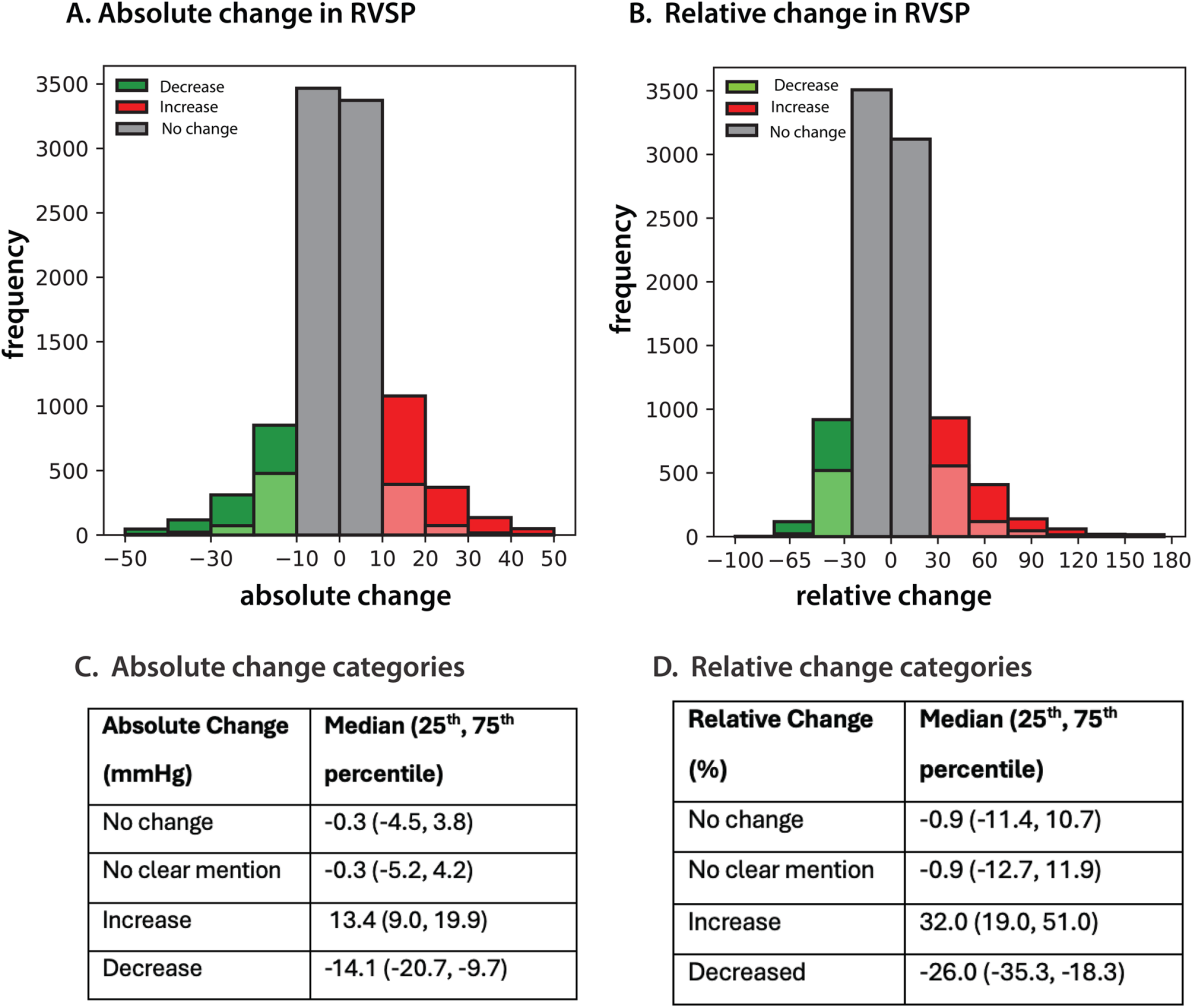
Distribution of (**A**) absolute change (**B**) relative change. The median distribution of absolute change (**C**), relative change (**D**). RVSP: right ventricular systolic pressure

Table 2 summarizes physician reporting for change in RVSP while Fig. 2 shows the distribution of the absolute change (Fig. 2-A) and relative changes (Fig. 2-B). The.

**Table 2:** lists and numbers of terms used for the classification of change

Classifiers	Examples of terminology used	*Number (percentage)
No change	No change, no significant change, unchanged, no significant difference	15,901(48.9%)
No clear mention of RVSP (indeterminate)	The comparative statement specifically refers to a change in another variable but does not mention RVSP (e.g. LVEF has improved).	4,766 (27.5%)
Increase	Increase, significant increase, deteriorated, Elevated, worsened	4,092 (12.5%)
Decrease	Decrease, reduced, improved	3,200 (9.9%)
Possible change statement	Descriptor words such as: possible change, slightly	48 (0.1%)
Reporting not consistent with numerical value change	The conclusion does not match the quantitative value. Either the physician disagrees with baseline reading or different comparator	330 (1.0%)
Status change comment only	Status post (s/p)	4 (0.01%)

*N = 32,656 echoes

LVEF: left ventricular ejection fraction; RVSP: right ventricular systolic pressure

The median and interquartile range for each change category (decrease, increase, no change, and indeterminant) are shown in Figs. 2-C and 2-D, respectively. Comparing the qualitative change with measured value change, it was verified that the “no change” category followed a normal distribution centered around 0. The median value for absolute was − 0.3 mmHg and − 0.9% for relative change. The indeterminate category also followed a normal distribution with median absolute change − 0.3 mmHg and relative change − 0.9%. In the “increase” category, the median absolute change was estimated at 13 mmHg, whereas the median relative change was 32%. In the “decrease” category, the median absolute change was estimated at 13 mmHg, too, while the median relative change was 26%.

#### Decision tree and gini index

**Fig. 3:**
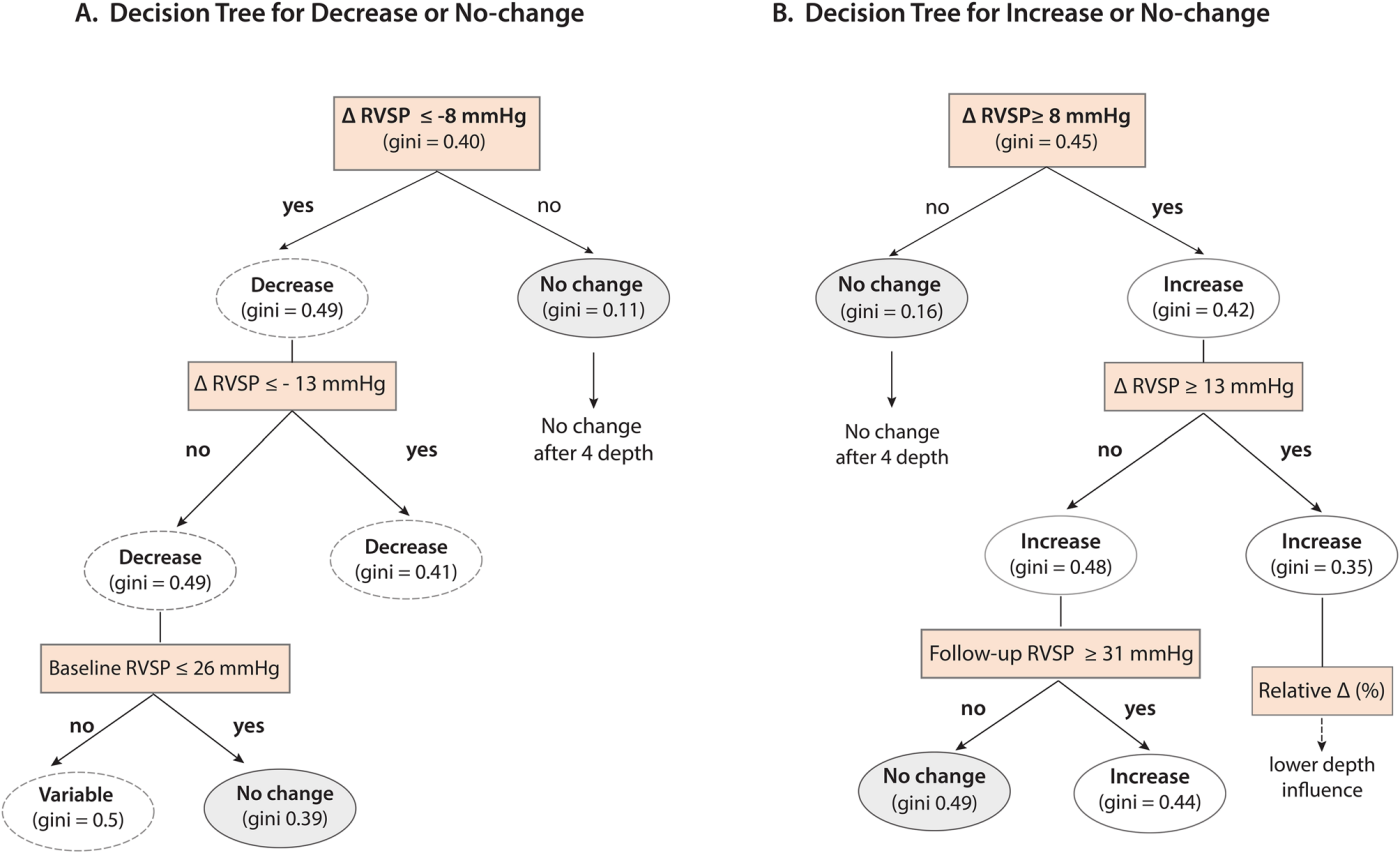
The summery of decision tree based on CART analysis. (**A**) Focused on “decrease vs. no change”. (**B**) Focused on “increase vs. no change”. Gini: gini impurity index; RVSP: right ventricular systolic pressure

Figure 3 shows the simplified CART decision tree for physician readings with the complete rendering shown in Additional file 2. A symmetry was observed for the increase and decrease tree structures. For both, the root node consisted of absolute change in RVSP of 8 mmHg. When RVSP increased or decreased by more than 8 mmHg (absolute value), a change was usually reported unless it occurred within the reference limits. With smaller changes, no significant change was usually reported.

More specifically, Fig. 3-A summarizes the decision tree analysis focused on the “decrease vs. no change.” If RVSP decreased by less than or equal to 8 mmHg, the decision tree classified to “no change” decision; if the absolute RVSP decrease was between 8 and 13 mmHg, the classification was further nuanced based on whether the baseline RVSP was estimated less than or equal to 26 mmHg. The decision tree made a “no change” decision if delta RVSP was less than 13 mmHg, in the context of baseline RVSP less than or equal to 26 mmHg. Relative change played a role in further layers of decision-making. Figure 3-B summarizes the decision tree analysis focused on “increase vs. no change.” The root node consisted of an absolute change of ≥ 8 mmHg, the first rule to split the classes. If the absolute RVSP change was <8 mmHg, the decision tree classified “no change”; if the absolute RVSP change was higher than 13 mmHg, the decision tree decided on “increase”. If the absolute RVSP increase was between 8 and 13 mmHg, the classification was further nuanced in the context of the following RVSP estimated value of 31 mmHg, or higher. If delta RVSP (absolute change) was less than 13 mmHg, the decision tree still decided that RVSP is “increased”, if the following RVSP value is estimated to be higher than or equal to 31 mmHg Relative change played a role in further layers of decision-making.

#### Test performance and cross-validation

The decision tree test performance was evaluated by accuracy, precision, recall, and presenting the F1-score using stratified cross-validation. In the “increased” model, the accuracy was 0.83 (95% CI: 95% CI: 0.80–0.84), precision 0.75 (95% CI: 0.73–0.76), recall 0.84 (95% CI: 0.80–0.84), and the F1-score was 0.79 (95% CI: 0.78–0.81). In the “decrease” model, the accuracy was 0.81 (95% CI: 0.80–0.83), precision 0.67 (95% CI: 0.65–0.68), recall 0.85 (95% CI: 0.83–0.87), and the F1-score was 0.74 (95% CI: 0.72–0.75).

We also did manual extraction of *n* = 27 randomly selected cases within classes of “increased”, “decreased” and “no change”, to validate the NLP classification by a level three cardiologist (F.H.) in our core lab. We used Cohen’s kappa (*k*) test to present the agreement which is mathematically described as: *k*= (P_o_ -P_e_)/(1-P_e_), when P_o_ is the observed agreement, and P_e_ is the expected agreement. The inter-reader agreement between NLP classification and core lab classification was *k* = 0.80, which reflects excellent agreement.

### Duplicate analysis and analytic variability

#### Cohort description

Table 3 presents the characteristics of the controls (*n* = 210) and the patient population with PAH (*n* = 208) selected for the analytical variability assessments. The PAH group was primarily female (77%), whereas the healthy cohort consisted of 46% females. The majority of the participants in the PAH cohort were White (59%), as were the controls (85%). Most of the PAH etiology was idiopathic (48%). The mean value for the measured MPAP in this group was 52 ± 14 mmHg, and the mean indexed pulmonary vascular resistance (PVRi) was 23 ± 11 mmHg.min.m^2^/L.

**Table 3:** Demographics and baseline right heart echocardiographic characteristics of the pulmonary arterial hypertension population

Characteristics	Control Median [IQR] *n* = 210	PAH Median [IQR] *n* = 208
Age(years)	57.9 [54.3–62.5]	46.8 [44.6–50.1]
Sex (male)	114/210 (54%)	48/208 (23%)
Race		
White	178/210 (85%)	123/208 (59%)
Asian	24/210 (11%)	21/208 (10%)
African American	4/210 (1.9%)	8/208 (3%)
Others	4/210 (1.9%)	69/208 (33%)
BMI (kg/m2)	24.4 [23.4–24.7]	28.8 [27.2–29.6]
SBP (mmHg)	119 [118–122]	115 [110–119]
DBP (mmHg)	74 [73–75]	72 [70–74]
HR (bpm)	61 [60–62]	80 [77–86]
Echocardiographic measures		
Peak TRV (m/s)	2.1 [2.05–2.2]	4.2 [4.04–4.3]
RV basal diameter(cm)	3.7 [3.6–3.8]	5.5 [5.4–5.6]
TAPSE (mm)	24.5 [24.0–25.8]	16.1 [15.0–17.0]
RAA max (cm^2^)	15.4 [14.7–16.1]	21.7 [20.6–23.7]
RVEDA (cm^2^)	21.6 [20.2–23.2]	36.9 [35.03–38.5]
RVESA (cm^2^)	13.0 [12.4–13.8]	30.0 [28.2–31.1]
RVFAC (%)	39.7 [38.9–40.3]	19.2 [17.7–20.1]
LVEF (%)	62.4 [61.8–63.3]	64.6 [63.1–65.5]

BMI: body mass index; DBP: diastolic blood pressure; HR: heart rate; LAVI: left atrial volume index; LVEF: left ventricular ejection fraction; LVID: left ventricular internal diameter; SBP: systolic blood pressure; RA: right atrium; RAA: right atrial area; RV: right ventricular; RVEDA: right ventricular end-diastolic area; RVESA: right ventricular end-systolic area; RVFAC: right ventricular fractional area change; TAPSE: tricuspid annular plane systolic excursion; TRV: tricuspid regurgitation velocity

#### Technical (analytic) variability

**Fig. 4:**
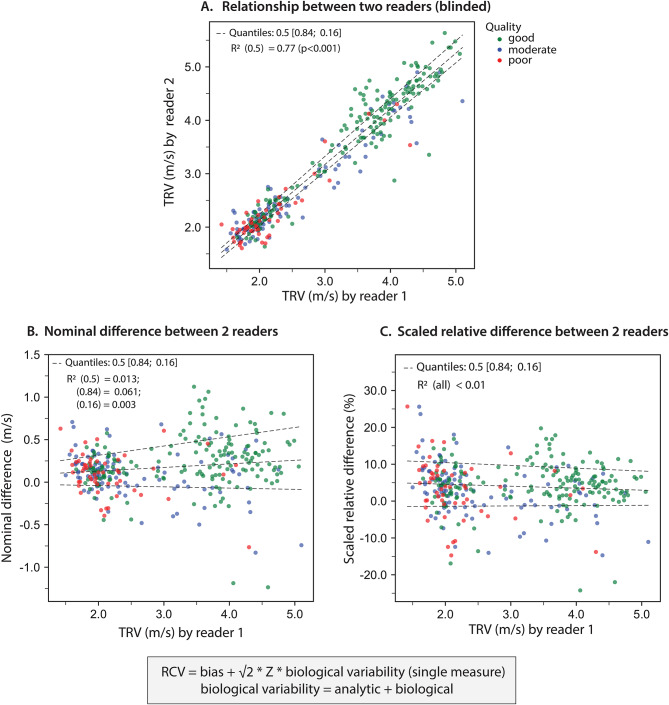
The analytical variability of tricuspid regurgitation velocity between two readers. (**A**) The relationship between two readers in three quantiles (50%, 16%, 84%), (**B**) the absolute differences, and (**C**) scaled relative differences showing precision and bias. RCV: reference change value; RVSP: right ventricular systolic pressure; Z: z-score

**Fig. 5:**
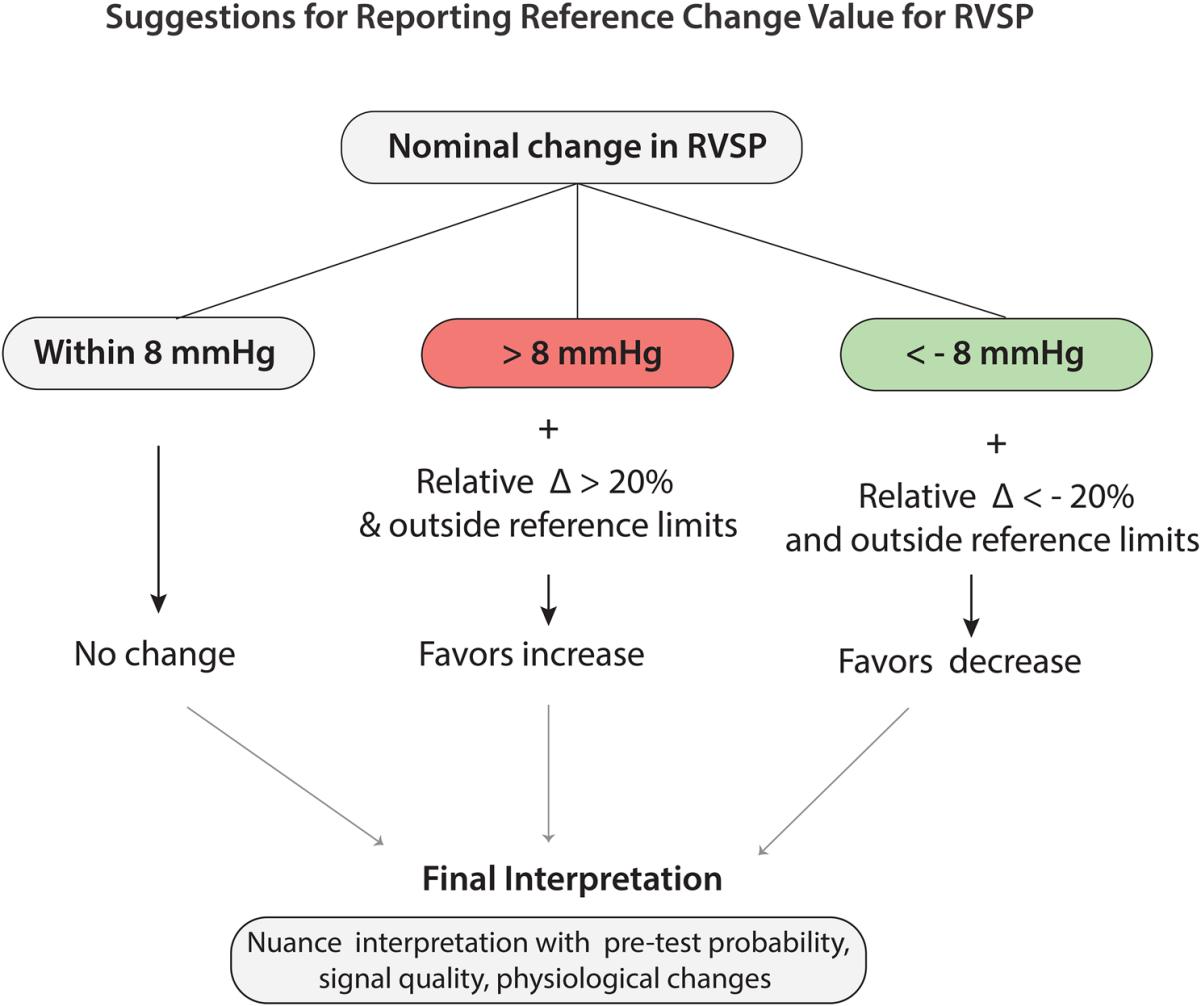
The figure summary demonstrates the consideration of analytical and biological variability when reporting reference changes. RVSP: right ventricular systolic pressure

Most patients in the PAH cohort had preserved LVEF, with a mean value of 64.6 ± 7.8%. Table 3 shows the echocardiographic right ventricular (RV) functional metrics. The TRV signal quality was good/excellent in 192 (44%), moderate in 118 (27%), low in 84 (19%), and without consensus in 40 (9.2%) of the measurements. Figure 4 highlights the association between RVSP readings (A) and the absolute (B) and relative variability (C) across the measurement range. The two measurements were closely related, with a correlation coefficient of 0.94, *p* < 0.001. The absolute median bias was 3.17 mmHg (2.72 to 3.84 mmHg) with a robust scaled precision of 4.56 mmHg (3.95 to 5.37 mmHg) (bootstrap values). The relative median bias was 10.23 (8.68 to 11.22) % with a robust scaled precision of 10.23 (8.68 to 11.22) %. The additional file 3 presents duplicate analysis metrics using parametric and robust methods. Based on the CVA of 8.9% (lowest confidence) and assuming a very optimistic within-subject biological variation of 4% (Crnko et al. [16] for patients in HF), the relative RCV for reporting meaningful change would be at least 20% (Fig. 5).

#### Clinical algorithm for reporting of RCV of RVSP

The study results led to clinical implementation changes in our laboratory to avoid overreporting changes in RVSP after verifying the acquisition of good-quality signals. This is summarized in Fig. 5, where absolute and relative changes in RVSP are combined to determine whether changes occur within the reference limits. Clinicians further consider the clinical context (pre-test probability) and associated changes (for example, in chamber size and function) to nuance the interpretation.

## Discussion

Determining meaningful clinical change is critical for evaluating patient stability and therapeutic response. Our study has two key findings. First, CART analysis revealed that physicians primarily base their conclusions on whether RCV in RVSP occurred based on absolute change and on whether these changes occur outside reference limits. Second, we found that the level of technical (analytic) variation is often higher than what is typically considered in clinical practice and could lead to over-reporting of change in RVSP. More importantly, our study invites future work on reference change value of RVSP and change in RV size and function in pulmonary hypertension and heart failure [17].

Our study used natural language processing to analyze the reporting of change in RVSP. While various terminologies were used, most could be easily interpreted as synonyms. However, in fewer than 1% of cases, ambiguous terms like “might” and “possibly” were encountered. Additionally, changes in RVSP were not explicitly mentioned in more than 20% of cases. Although this generally aligned with situations where no significant change was observed, it could also represent cases where meaningful changes were potentially overlooked.

CART analysis is a straightforward yet powerful tree-based analytical tool for identifying influential variables and critical thresholds for classification. CART has been employed in various cardiovascular imaging studies. For instance, Potter et al. demonstrated its utility in integrating clinical risk scores with diastolic parameters to predict heart failure incidence [12]. Similarly, Schilling et al. applied CART to define thresholds and hierarchies in funding decisions [18]. We used CART analysis to explore the thresholds for reporting longitudinal changes in RVSP. We separated the analysis into “increase” and “decrease” of RVSP, limiting the tree depth to four levels to ensure streamlined decision trees. We included the latest RVSP value in the increase model to capture changes within the reference range. The “decrease” model used the baseline RVSP to assess changes within the reference limits. Our analysis revealed that physicians at our institution tended to rely on absolute changes (~ 8 mmHg) and considered whether the changes fell within reference limits. Relative changes played a role in decision-making and were observed at a lower depth in the trees. The test performance, assessed through cross-validation, demonstrated strong accuracy (> 0.8), suggesting that the model effectively captured a significant portion of the reporting practices. No prior study has utilized decision tree analysis to investigate physician reporting and gain insights into RCV in imaging studies.

While CART analysis provides insights into how physicians report changes, it does not address whether these changes align with technical (analytic) and biological variation [19]. RCV integrates both sources of variation, offering a more robust framework for interpreting serial measurements. In laboratory medicine, extensive analytic and biological variation data are available for circulating blood biomarkers as reliable references for calculating RCV. In contrast, similar data for imaging studies are not well established. The second part of our study focused on providing insights into the potential sources of variation for RVSP. We employed a robust methodology for analytic variability based on robust precision. Compared to the standard mean bias or root mean square coefficient of variation (CV), this approach, proposed by Hyslop et al., is less susceptible to outliers and effectively separates bias from precision [20]. We observed 8.9% scaled percentile precision between two core laboratory readers with a slight systematic difference (bias). Several factors may contribute to analytic variability between two readers, including the selection of the signal and the choice of interface for the estimation of modal frequency. Additionally, on reacquisition, variations in the insonation angle, with errors depending on the angle’s cosine, and the signal’s scale can also influence inter-reader variability.

Data on biological variability in RVSP signals is even more challenging to find [16, 20–22]. In a small but insightful study, Cnko et al. examined ten heart failure patients implanted with CardioMEMS sensors [16]. They found that the total CV for morning readings, including biological and analytic variation, ranged from 3.4% to 16.8% [16]. The average variation was even more significant in the evening. Although small, this study highlights the considerable variability in pressure measurements. Changes in cardiac output, pulmonary capillary wedge pressure, or fluctuations in neurohormonal regulation may partially explain this variability. An approximate linear relationship between MPAP and cardiac output is observed in the pulmonary circulation. In-silico studies integrating advanced imaging techniques such as phase-contrast cardiac MRI with computational modeling have provided valuable insights into ventricular flow dynamics, emphasizing the potential of such approaches in understanding intracardiac hemodynamics [23, 24]. In addition, filling pressure can also influence the extent of biological variation. Boerrigter et al. demonstrated that in patients with COPD, those with elevated right atrial filling pressures exhibited smaller respiratory-induced changes in PA pressure [25]. This suggests that right-heart filling dynamics may influence PA responses, underscoring the importance of considering right atrial pressure when evaluating RVSP variability.

Our results underscore the importance of variability in measures. Assuming an 8% analytic variability and a 4% biological variation (optimistic stability) [16], the total CV would be around 8.9%, with an RCV of 20–25%, depending on the Z value and pre-test probability of change. Combining absolute and relative change is a simple way to account for a CV (%) variation that can change over the range of values (with higher percent variation in the lower range). This led to the simple algorithm implemented in our laboratory to use 8–10 mmHg for an absolute change of at least 20% relative change. Although conservative, this accounts for the lower biological variation with severe disease (Fig. 4-A). Of course, this should always be placed in the context of other changes in ventricular function and hemodynamics. Lastly, the role of automated segmentation and artificial intelligence (AI) in estimation for RVSP is emerging, which can lead to improved technical variability, although this will not correct technical variability as found by a recent study by Celestin et al [26].

The study has several limitations. First, although the sample size is large, it represents only a subset of our entire database. Despite the large number of RHC structured data, the RVID and TAPSE measurements were limited to certain numbers of patients. Additionally, physician interpretation of prior studies was not accounted for, and the limited data on biological variation in the literature constrained our theoretical modeling. Future studies should validate these findings in an external population, especially comprehensive research into biological variability of RVSP would be insightful.

## Conclusions

Our analysis of physician reporting of RVSP provides valuable insights for reporting change in RVSP. Combining absolute changes of 8 mmHg, relative change of 20%, and whether the changes occur in the reference limits represents a practical way to avoid over-reporting change in the clinical laboratory. Future studies should validate these thresholds through outcome analysis integrate deep learning methods for analysis, and incorporate echocardiographic readings within the clinical context.

## Electronic Supplementary Material

Below is the link to the electronic supplementary material.

Supplementary Material 1

## Data Availability

The data set generated and analyzed during the current study is not publicly available due to the sensitive and protected nature of healthcare information; however, it is available from the corresponding author upon reasonable request.

## Abbreviations

ASE: American society of echocardiography
ESC/ ERS: European Society of Cardiology/ European Respiratory Society
CART: Classification and regression tree
CL: Core laboratory
CO: Cardiac output
CV: Coefficient of variation
LVEF: Left ventricular ejection fraction
MPAP: Mean pulmonary arterial pressure
PA: Pulmonary artery
PAH: Pulmonary arterial hypertension
PASP: Pulmonary arterial systolic pressure
PH: Pulmonary hypertension
PVR: Pulmonary vascular resistance
RAP: Right atrial pressure
RCV: Reference change value
RHC: Right heart catheterization
RV: Right ventricle
RVSP: Right ventricular systolic pressure
SD: Standard deviation
TRV: Tricuspid regurgitation
TTE: Transthoracic echocardiogram
