# Supplementary material for: Reporting changes in right ventricular systolic pressure: insights from Classification and Regression Tree (CART) analysis
Source: Echo Res Pract. 2026 Jun 8;13:19. doi: 10.1186/s44156-026-00120-8 (PMC13244848; doi:10.1186/s44156-026-00120-8)
Supplement: Supplementary file 1 — Supplementary Material 1 [file 44156_2026_120_MOESM1_ESM.docx]

**Additional file 1.** Likert scale for quality grading system of Tricuspid regurgitation Doppler signal

| **Grading** | **Completeness** | **Modal frequency** | **Simplified Grading** |
| --- | --- | --- | --- |
| 5 | Complete | Well defined modal frequency | Good |
| 4 | Complete | Modal frequency not as well defined |  |
| 3 | Peak not perfectly defined | - | Acceptable |
| 2 | Peak not visualized but transitions visualized | - | Poor |
| 1 | Neither peak or transition visualized | - |  |

**Additional file 2.** (A) The summary of the decision tree analysis focused on “decrease vs. no change” in RVSP. (B)The completed version of the decision tree output focused on “increase vs. no change” in RVSP.

**Additional file 3.** Showing the details of the precision and bias analysis between two core lab readers.

| **Type of change** | **Variable name** | **Sample Value** | **Bootstrap Value** | **95% CI** |
| --- | --- | --- | --- | --- |
| Absolute | Mean bias | 5.06 | 5.04 | 4.16 to 5.90 |
|  | Median bias | 3.16 | 3.17 | 2.72 to 3.84 |
|  | Lower limit agreement | -9.15 | -9.15 | -12.63 to -6.89 |
|  | upper limit agreement | 30.69 | 30.64 | 23.21 to 32.11 |
|  | Percentile precision | 4.56 | 4.56 | 3.95to 5.37 |
|  | RMS CV | 7.23 | 7.20 | 6.40 to 8.03 |
| Relative | Mean relative bias | 9.42 | 9.38 | 7.89 to 10.94 |
|  | Median relative bias | 10.32 | 10.23 | 8.68 to 11.22 |
|  | Lower limit relative bias | -28.07 | -28.07 | -33.25 to -18.29 |
|  | upper limit relative bias | 38.50 | 38.50 | 34.58 to 44.24 |
|  | Percentile relative precision | 8.92 | 8.96 | 7.91 to 10.08 |
|  | RMS relative CV | 12.58 | 12.60 | 11.68 to 13.49 |
